# Adhesively Bonded Steel–Concrete Composite Structures: A Systematic Literature Review

**DOI:** 10.3390/ma19132804

**Published:** 2026-07-01

**Authors:** Alexandre Rocha, Isabel B. Valente, José B. Aguiar

**Affiliations:** 1 ISISE—Institute for Sustainability and Innovation in Structural Engineering, University of Minho, Campus de Azurém, 4800-058 Guimarães, Portugal; alexandrerochasalles@gmail.com (A.R.); isabelv@civil.uminho.pt (I.B.V.); 2 CTAC—Centre for Territory, Environment and Construction, University of Minho, Campus de Azurém, 4800-058 Guimarães, Portugal

**Keywords:** adhesive bonding, steel–concrete composites, structural behavior, bond behavior, interfacial mechanics, PRISMA 2020 review

## Abstract

Adhesively bonded steel–concrete composite structures have emerged as a promising alternative to conventional mechanically connected systems, offering continuous stress transfer and potential improvements in stiffness, load redistribution, and serviceability behavior. However, uncertainties regarding durability and long-term reliability have limited their broader implementation in structural practice. This study presents a PRISMA 2020-based systematic literature review synthesizing experimental, numerical, and theoretical research on adhesively bonded connections in steel–concrete composite structures. A total of 64 peer-reviewed journal articles published between 2000 and February 2026 were identified through systematic screening of Scopus and Web of Science datasets. The lack of methodological homogeneity across studies regarding testing protocols, conditions, and reported performance metrics in the literature led to a qualitative thematic synthesis and a bibliometric mapping. A total of six thematic axes (global behavior of bonded members, bond interface mechanics and characterization, numerical modeling approaches, strengthening and retrofit solutions, durability and long-term performance, and hybrid connection systems) were developed to encompass all the studies’ primary objectives, synthesize the most recurring themes, and highlight a significant gap related to durability and long-term performance in the state of the art. Beyond classifying the literature, this review contributes a substrate-conditioned interpretive framework that links interfacial mechanics, durability evolution, and reliability-based design, and it sets out a structured roadmap towards durability-informed design criteria for adhesively bonded steel–concrete composite systems.

## 1. Introduction

Steel–concrete composite structures are widely employed in civil engineering due to their efficient integration of complementary material properties, combining steel’s tensile resistance with concrete’s compressive capacity [1,2,3]. Composite action is traditionally achieved through discrete mechanical connectors, such as headed studs or bolted systems, which enable shear transfer between steel and concrete elements [4,5]. Although mechanically connected systems are well established in structural design practice, their discrete nature may introduce stress concentrations, localized slip, and fatigue-sensitive regions that influence serviceability performance and long-term behavior [6].

Adhesive bonding has emerged as a potential alternative or complementary strategy for achieving composite action in steel–concrete systems [7]. By providing continuous stress transfer along the interface, adhesive layers can reduce localized stress concentration and promote more uniform interaction between materials [8,9]. Experimental investigations have reported improvements in initial stiffness, delayed interfacial slip, and enhanced load redistribution under monotonic loading conditions [7,10]. These advantages have stimulated growing academic interest, particularly in strengthening and reinforcement applications, where geometric constraints or constructability considerations may limit the use of conventional connectors. Much of the early understanding of bonded reinforcement derives from the externally bonded reinforcement tradition, where bond and debonding behavior at the composite-to-concrete interface has been extensively characterized [11], and these insights provide a conceptual foundation for the adhesively bonded steel–concrete systems examined here.

Despite these promising short-term structural benefits, the long-term performance of adhesively bonded steel–concrete composite systems remains insufficiently understood. The adhesive interface is inherently sensitive to environmental exposure, including moisture ingress, thermal variations, and aggressive chemical agents such as chlorides [12,13]. In structural applications, these environmental actions frequently act simultaneously with sustained or cyclic mechanical loading, potentially leading to progressive degradation of bond strength, stiffness, and composite action [7]. As a result, structural efficiency observed under controlled laboratory conditions may not accurately represent structural behavior throughout the intended service life.

The existing body of research is characterized by significant methodological diversity. Studies range from small-scale interfacial characterization tests and push-out experiments to bending tests of composite members and advanced finite-element modeling incorporating interface and cohesive-zone formulations. While these investigations provide valuable insight into bond mechanics and structural response, they often focus on isolated performance indicators or short-term loading scenarios. Durability-related research remains comparatively limited and is frequently restricted to single environmental exposure mechanisms, with little integration of environmental–mechanical coupling or time-dependent degradation modeling. Consequently, a consolidated framework linking structural efficiency, degradation mechanisms, and long-term reliability is currently lacking.

Given the recent expansion of research activity and the absence of durability-informed design criteria, a structured synthesis of the available evidence is necessary to clarify the state of knowledge and identify unresolved challenges. A systematic literature review provides a transparent methodology for mapping dominant research themes, methodological trends, and performance assessment strategies, while also revealing critical research gaps. This study, therefore, presents a PRISMA 2020 [14] systematic review of adhesively bonded steel–concrete composite structures, focusing on structural efficiency and durability. The primary objective is to critically synthesize scientific evidence on the structural behavior of adhesively bonded steel–concrete systems. The review aims to identify how structural behavior is quantified, which assessment methods are employed, how this structural solution is discussed in the literature, the current state of the art regarding its potential large-scale use, and its limitations.

Ultimately, the key question guiding this review is the following: To what extent does the current body of evidence support the development of reliability-based design frameworks for adhesively bonded steel–concrete composite systems?

By addressing this question, this review aims to provide a structured overview of current research, clarify the relationship between structural efficiency and service life, and outline future research directions to develop reliable design approaches for adhesively bonded steel–concrete composite structures. In contrast to earlier reviews that primarily catalog strengthening techniques or summarize reported bond-strength results, the specific contribution of this work lies in articulating a substrate-conditioned framework that connects interfacial mechanics, durability evolution, and long-term reliability, and in translating that framework into a roadmap towards reliability-based design, rather than treating adhesive bonding as an exclusively experimental strengthening technique.

The paper is structured as follows: Section 2 describes the methodology; Section 3 presents and analyzes the results; Section 4 discusses the main findings, limitations, and integrated performance framework; while Section 5 summarizes the conclusions and future research priorities.

## 2. Methodology

This study employs a systematic literature review (SLR) methodology in accordance with the PRISMA 2020 (Preferred Reporting Items for Systematic Reviews and Meta-Analyses, version 2020) [14] guidelines that ensure transparency, reproducibility, and methodological rigor as presented in the PRISMA checklist provided as Supplementary Material (File S1). PRISMA-based systematic reviews are increasingly adopted in construction and structural engineering to enhance transparency and reproducibility, particularly in emerging or multidisciplinary domains, such as risk management in green buildings, robotic construction technologies, and fiber-reinforced strengthening solutions [15,16,17].

The review focuses on the structural efficiency and durability of adhesively bonded steel–concrete composite structures.

**Figure 1:**
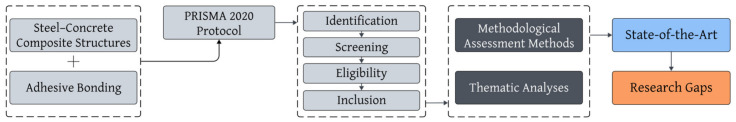
Summary of the systematic literature review (SLR) process.

The overall methodological workflow is illustrated in Figure 1, which summarizes the PRISMA-based study selection process and the thematic synthesis framework adopted in this review. The process comprises four sequential stages: identification, screening, eligibility, and inclusion, followed by structured data extraction and quantitative and qualitative synthesis. In the identification stage, records are retrieved from the selected databases using the predefined search strategy, and duplicate entries are removed prior to further assessment. During the screening stage, the retrieved records are evaluated at the title and abstract level to exclude studies clearly outside the scope of the review. In the eligibility stage, the full texts of potentially relevant studies are assessed against the predefined inclusion and exclusion criteria, and exclusions are documented with explicit reasons. Finally, in the inclusion stage, studies satisfying all eligibility requirements are retained and incorporated into the synthesis.

The review adopts a qualitative and descriptive approach, integrating comparative and thematic analyses. Rather than applying a formal quantitative meta-analysis or a standardized risk-of-bias tool, as commonly done in clinical and biomedical PRISMA-based reviews, this study derives critical insights through a systematic cross-study comparison of research objectives, methodological strategies, performance indicators, and reported limitations. This approach is justified by the substantial heterogeneity of experimental configurations and reporting practices across the included studies, which precludes meaningful statistical aggregation. The output of this comparative process is a structured thematic synthesis that maps the current state of evidence, identifies dominant research themes and recurring methodological patterns, and highlights critical knowledge gaps in the structural efficiency and durability assessment of adhesively bonded steel–concrete composite systems.

For clarity, the methodological parameters used in this review were defined at five complementary levels. First, the search parameters comprised the databases consulted, the search fields, the Boolean query, the publication period, the language, and the document type. Second, the eligibility parameters defined the structural system, load-transfer mechanism, and boundary conditions for inclusion or exclusion. Third, the screening parameters specified the sequence of identification, duplicate removal, title-and-abstract screening, full-text eligibility assessment, and final inclusion, as illustrated in Figure 1 and quantified in the PRISMA flow diagram presented in Section 3.1. Fourth, the extraction parameters defined the bibliographic, methodological, material, interface, testing, modeling, and outcome information recorded for each study. Finally, the appraisal parameters defined the domains used to assess methodological quality and reporting bias. These parameters are operationalized through the eligibility criteria and decision rules for boundary cases in Section 2.1, as well as the risk-of-bias descriptors in Section 2.7.

### 2.1. Eligibility Criteria

To ensure the relevance and consistency of the selected studies, a set of inclusion and exclusion criteria was defined prior to the screening process, in accordance with PRISMA 2020 recommendations [14]. These criteria were established to guarantee that only studies directly addressing adhesively bonded steel–concrete composite systems were retained for analysis. The eligibility criteria applied in this review are summarized in Table 1.

Because the general criteria in Table 1, by themselves, do not resolve several recurrent boundary cases, a complementary set of operational decision rules was applied during full-text assessment. The governing principle across all cases was the primacy of the adhesive bond in load transfer, that is, whether the bonded interface constituted the principal mechanism of composite action rather than an auxiliary feature. Four categories proved particularly sensitive to this distinction and were treated as follows. Steel-plate strengthening of reinforced concrete members was retained only when the adhesive bond constituted the primary shear-transfer mechanism and was excluded when bonding was auxiliary to mechanical anchorage, such as bolts or anchors. Hybrid FRP and steel strengthening configurations were excluded unless a steel–concrete adhesive interface was explicitly characterized, because otherwise the load-transfer behavior belongs to the FRP-to-concrete bonding tradition. Adhesive-assisted mechanical connectors were retained and assigned to the hybrid connection axis, given that the adhesive contributes measurably to the connection response. Finally, material-scale bond tests were included only when the reported results were explicitly linked to a structural joint configuration, and excluded when the work remained at the level of coupon or interface characterization without structural interpretation. These rules are consolidated in Table 2.

### 2.2. Information Sources

A comprehensive literature search was conducted using Scopus and Web of Science, with recent systematic reviews in the construction and engineering domains, employing dual-database strategies to enhance coverage and reduce selection bias [16,18,19]. The final search was performed in February 2026.

Only peer-reviewed journal articles written in English were considered. The inclusion period was from January 2000 to February 2026, and it was used to capture the full evolution of research on adhesively bonded steel–concrete composite systems.

To ensure completeness, the reference lists of all studies retained after full-text screening were manually examined to identify additional relevant publications not captured through database searching.

### 2.3. Search Strategy

The search strategy was applied to the title, abstract, and keyword fields of each database. The query combined core terms representing the three essential components of the investigated system: the concrete substrate, the steel element (including cold-formed steel, CFS), and the adhesive bonding mechanism, resulting in a Boolean structure as follows:

**(“concrete”) AND (“steel” OR “CFS”) AND (“adhesive” OR “bonded”)**

These Boolean operators were intentionally formulated to ensure that retrieved studies explicitly addressed composite systems in which concrete and steel are connected through an adhesive interface. By requiring the simultaneous presence of material and bonding descriptors, the search string maximized thematic relevance while minimizing the inclusion of studies focused solely on material-scale adhesive research or mechanically connected composite systems. The deliberately concise structure of the query was adopted to preserve transparency and reproducibility while maintaining high precision within the field’s established terminology. Even so, a broader set of synonyms could broaden recall, and to test this, a complementary sensitivity search incorporating a representative set of coherent synonyms was performed in both databases, using the broadened string (“concrete”) AND (“steel” OR “CFS”) AND (“adhesive” OR “bonded” OR “bonding” OR “epoxy” OR “glued” OR “resin” OR “bond line” OR “interface”). This returned 8661 non-duplicated records, compared with 4541 for the core query. After title and abstract screening, 148 records were assessed at full text, and 64 met the eligibility criteria, all of which were already included in the corpus assembled from the core search and its backwards-citation screening. The broadened query thus identified no additional eligible study; the records it added were predominantly concrete–steel interface investigations without an adhesive bond, indicating that wider synonym coverage increased noise rather than recall. Consistent with the PRISMA 2020 framework [14], the systematic character of this review derives from a prespecified, transparent, and reproducible protocol, combined with supplementary citation searching, rather than from maximal lexical recall within a single Boolean query.

### 2.4. Selection Process

During the identification stage, records were retrieved from the Web of Science, which is a multidisciplinary citation database that includes over 240 million records and billions of cited references, making it one of the most comprehensive citation resources available. Scopus, a major multidisciplinary abstract and citation database, currently covers approximately 100 million records worldwide and encompasses peer-reviewed literature across disciplines [20,21]. Duplicate entries were removed using reference management software to ensure data quality before bibliometric analysis. The remaining records underwent title and abstract screening to exclude studies clearly outside the scope of the review.

Full-text articles considered potentially relevant were then assessed against the predefined inclusion and exclusion criteria, as summarized in Table 1. The screening and eligibility assessment were conducted independently by the authors, each applying the predefined inclusion and exclusion criteria to titles, abstracts, and full texts. Disagreements between reviewers were resolved through discussion involving all three authors until consensus was reached. This dual-reviewer approach was adopted to minimize selection bias and ensure methodological transparency, in accordance with PRISMA 2020 recommendations [14].

The final number of studies included in the systematic review, along with the number of records excluded at each stage and the reasons for exclusion at the full-text level, are presented in the PRISMA flow diagram (Section 3), which provides a detailed summary of the selection process.

### 2.5. Data Collection Process

A structured data extraction process was applied to all studies included in the final corpus. The extracted information was systematically recorded to ensure consistency across studies and to support a transparent synthesis aligned with the research questions. For each selected article, the following data were collected: authorship, title, keywords, abstract, publisher (journal title), publication year, Digital Object Identifier (DOI), and number of citations. Data extraction was conducted manually using the selected databases, and no automation tools were employed. Authorship and journal title were extracted as bibliographic descriptors only and were not used as eligibility criteria during screening or full-text assessment.

### 2.6. Data Items

Within the selected group, a critical analysis was conducted of the type of steel–concrete composite system and structural configuration, the adhesive type and bonding technique, the experimental and/or numerical assessment methods, the performance indicators used to evaluate structural behavior, the long-term behavior evaluation condition (if applicable), and key findings related to structural behavior and limitations. Given the heterogeneity of testing protocols, conditions, and performance metrics reported in the literature, a qualitative thematic synthesis was adopted rather than a quantitative meta-analysis.

#### Outcomes

Considering the qualitative thematic synthesis to be applied, the collected data were organized and analyzed within six shared themes: global behavior of bonded members, bond interface mechanics and characterization, numerical modeling approaches, strengthening and retrofit solutions, durability and long-term performance, and hybrid connection systems. This thematic organization enabled a comparative evaluation of diverse approaches and research outcomes across studies while maintaining coherence with the review’s objectives.

### 2.7. Study Risk of Bias Assessment

The risk of bias of the included studies was assessed using a structured methodological quality framework specifically adapted to experimental and numerical research in structural engineering. Given the absence of a universally accepted bias assessment tool for engineering studies, a domain-based evaluation approach was developed to ensure consistency and transparency.

The assessment considered the following domains:

- D1—Clarity and completeness of experimental design description;
- D2—Adequacy of specimen characterization, including material properties and geometry;
- D3—Transparency in reporting adhesive properties and surface preparation procedures;
- D4—Sample size and replication strategy;
- D5—Control of testing conditions, including loading protocol and environmental exposure;
- D6—Reporting of statistical dispersion measures;
- D7—Consistency between stated methodology and reported results;
- D8—Validation procedures in numerical studies, when applicable.

No automation tools were used in the risk-of-bias assessment process. For each domain, studies were classified as presenting low risk of bias, some concerns, or high risk of bias, based on predefined qualitative criteria. The results of the risk of bias assessment were used to support the interpretation of the findings and to identify methodological limitations in the existing body of literature, as presented in Section 3.3 and Section 3.5.

The qualitative criteria applied to classify each domain as low risk, some concerns, or high risk are summarized in Table 3. The overall study-level judgment was then derived from the domain ratings using a predefined rule. A study was classified as high overall risk of bias when two or more domains were rated high risk, or when a single domain was rated high risk, together with two or more domains rated as some concerns. A study was classified as having low overall risk of bias only when no domain was rated high risk, and at most one domain raised some concerns. All remaining configurations were classified as moderate overall risk. Final overall judgments were reached by consensus among the three authors.

### 2.8. Effect Measures

As this review aimed to provide a structured comparative and descriptive analysis of the existing literature rather than a quantitative synthesis of structural behavior results, no statistical effect measures were calculated.

Reported outcomes were presented as described in the original studies, and comparisons were conducted at a qualitative and conceptual level. When numerical values were available, they were discussed descriptively without calculation of pooled effect sizes or standardized differences.

The synthesis focused on identifying research trends, methodological approaches, thematic developments, and recurring performance patterns across studies, rather than deriving aggregated quantitative metrics.

### 2.9. Synthesis Methods

#### 2.9.1. Eligibility for Each Synthesis

All 64 studies retained following the full-text eligibility assessment were considered eligible for inclusion in the thematic synthesis. No additional eligibility criteria were applied at the synthesis stage, as the predefined inclusion criteria were sufficient to define a coherent and thematically bounded analytical corpus. All included studies were examined within a unified qualitative framework encompassing six thematic axes, as detailed in Section 2.6. Each study was assigned to a primary thematic axis based on its stated research objective, as reported in the original publication. When a study addressed more than one theme, classification was determined by the principal focus, with secondary themes noted when relevant. This classification process defined the analytical structure for the comparative synthesis presented in Section 3.4 and ensured that all included studies contributed to the evidence base without being selectively excluded at the synthesis stage. The combination of bibliometric mapping and thematic classification has been successfully applied in recent systematic reviews in the construction domain to identify research clusters, emerging themes, and knowledge gaps [16,19].

Numerical or analytical models were therefore not selected a priori as candidate design models, nor were specific modeling approaches imposed as an inclusion criterion. Instead, studies containing numerical, analytical, or hybrid experimental-computational modeling were retained only when they satisfied the same eligibility criteria applied to the full corpus. After inclusion, the modeling approaches reported by the original authors, including finite-element formulations, cohesive-zone models, bond-slip laws, parametric simulations, and analytical formulations, were extracted and classified descriptively. This procedure ensured that the review synthesized the numerical evidence available in the literature, rather than selecting or endorsing a particular numerical model.

#### 2.9.2. Data Preparation

Extracted data were cleaned and organized before synthesis to ensure consistency in terminology and classification. Duplicate records retrieved from the databases were removed before screening. Bibliographic and technical information was checked for consistency across the extraction matrix, and terminology was harmonized for methodological axes, thematic axes, loading types, failure modes, and environmental conditioning categories. No numerical transformations or statistical standardization procedures were applied. Missing information was not imputed; instead, it was recorded as “not reported” to preserve transparency regarding the reporting limitations of the original studies. The final classification of studies into methodological, thematic, and risk-of-bias domains was checked for internal consistency before bibliometric mapping and qualitative synthesis.

#### 2.9.3. Presentation of Results

Results were tabulated in structured comparative matrices that summarized study characteristics, thematic axes, and the primary objective. Descriptive statistics were employed to analyze publication trends over time, citation counts, publication venues, and the study’s methodological approach. Bibliometric mapping was conducted using VOSviewer version 1.6.21 [22] to analyze keyword co-occurrences, enabling the identification of thematic clusters, research hotspots, and recurring conceptual patterns. Bibliometric mapping was employed as a preliminary structuring tool, while thematic clustering was subsequently refined through manual expert-based classification, following approaches adopted in recent construction-focused systematic reviews [18].

No forest plots or pooled statistical diagrams were generated, as quantitative synthesis was not performed.

### 2.10. Reporting Bias Assessment

Reporting bias was qualitatively assessed to identify potential imbalances in the way study outcomes were presented across the included literature. Given the absence of effect-size aggregation and the heterogeneity of experimental designs, a statistical assessment of publication bias was not applicable.

The assessment focused on identifying patterns of selective outcome reporting, preferential publication of favorable structural behavior results, incomplete reporting of experimental parameters, and limited documentation of negative or neutral findings. Attention was given to the reporting of statistical dispersion measures, adhesive mechanical properties, surface preparation protocols, environmental exposure conditions, and failure mode descriptions.

The evaluation was conducted through a systematic comparison of reported outcomes and methodological transparency across studies. Observed patterns were considered during the synthesis of evidence and the interpretation of conclusions. The findings of the reporting bias assessment are presented in Section 3.5.

### 2.11. Certainty Assessment

A formal GRADE (Grading of Recommendations Assessment, Development, and Evaluation) assessment was not conducted because no quantitative synthesis was performed. However, a qualitative certainty assessment, informed by GRADE principles and adapted to the evidence from experimental structural engineering, was applied to two outcome domains: short-term structural efficiency and long-term durability performance.

## 3. Results

### 3.1. Search and Selection Process

Following the database search, a total of 4541 records were identified. After removal of duplicates, 2731 records remained for title and abstract screening. Of these, 123 articles were assessed for full-text eligibility, resulting in 64 studies included in the final synthesis. The main reasons for exclusion were the absence of one or more structural elements (concrete, steel, or adhesive), the use of adhesive joints that deviated from the functional bonding mechanism between concrete and steel, and non-structural applications.

**Figure 2:**
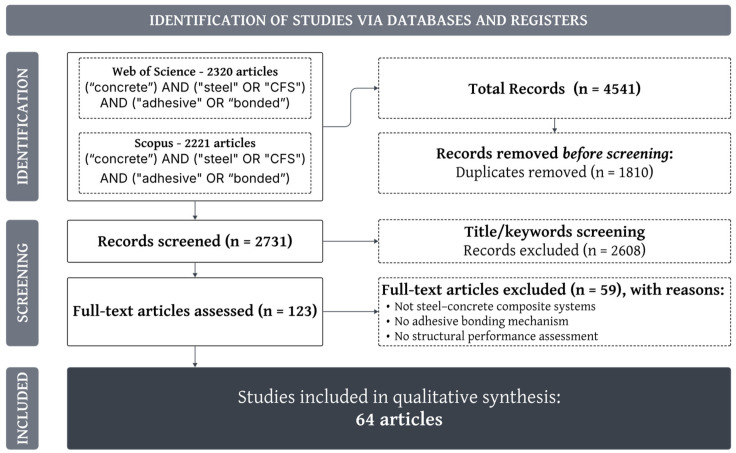
PRISMA 2020 flow diagram.

The study selection process is illustrated in the PRISMA flow diagram (Figure 2).

### 3.2. Study Characteristics

The methodological classification of the reviewed studies reveals a strong predominance of experimentally driven research. Table 4 presents the distribution of the literature across five defined methodological axes.

**Figure 3:**
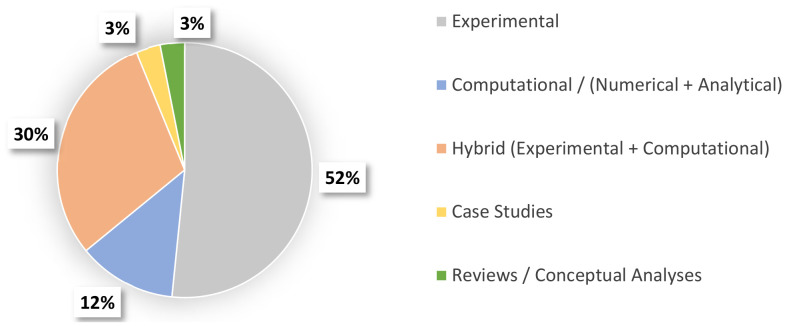
Numerical proportional distribution of studies within methodological axes.

The numerical distribution of the literature across five defined methodological axes is shown in Figure 3.

Experimental investigations account for 52% of the sample, indicating a research landscape strongly anchored in empirical validation. These studies rely primarily on laboratory-based testing, physical instrumentation, and direct measurement of structural or interfacial response. This predominance reflects the field’s emphasis on empirical validation, particularly in understanding material behavior, structural performance, and deterioration mechanisms under controlled conditions.

Hybrid approaches, combining experimental testing with numerical modeling, represent 30% of the sample. These studies typically use laboratory results to calibrate or validate computational models, enabling parametric extensions and predictive analyses. The significant proportion of hybrid studies indicates a progressive methodological integration aimed at improving robustness and applicability.

Purely computational or analytical investigations constitute 12% of the reviewed works. These contributions are generally based on FEM, parametric simulations, or analytical formulations, with experimental validation from the literature. Although smaller in scale, this axis plays a critical role in advancing predictive capabilities and exploring scenarios that may not be feasible to experiment with.

Case study research and review/conceptual analyses each represent only 3% of the total sample. The limited presence of large-scale structural applications and systematic synthesis studies suggests an important research gap, particularly in field validation and knowledge consolidation.

Overall, the results indicate a research domain strongly anchored in experimental validation, with emerging integration of numerical methods, yet with limited translation into large-scale real-world implementations or comprehensive conceptual syntheses.

#### 3.2.1. Publication Trends over Time

**Figure 4:**
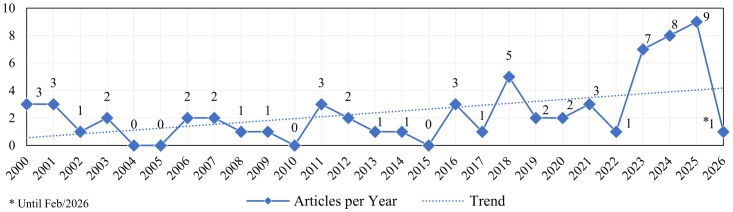
Annual distribution of publications included in the systematic review (from 2000 to February 2026).

To illustrate the evolution of scholarly interest in adhesively bonded steel–concrete composite systems, the annual publication distribution of the 64 studies included in this review is presented in Figure 4.

The temporal distribution reveals a relatively low research output during the early 2000s, with annual publications generally limited to one to three articles per year. This initial phase appears to correspond to foundational investigations primarily focused on interface behavior and proof-of-concept experimental validation.

A pronounced increase in publication frequency becomes evident between 2023 and 2025, a period when the annual number of publications reaches its highest levels in the dataset. This recent surge indicates an expansion phase in the field, reflecting intensified academic interest and broader methodological diversification.

This upward trend reflects growing research activity and intensified academic interest in recent years, associated with increased attention to durability performance, optimization strategies, and the development of advanced structural adhesives.

#### 3.2.2. Bibliometric Keyword Co-Occurrence Analysis

**Figure 5:**
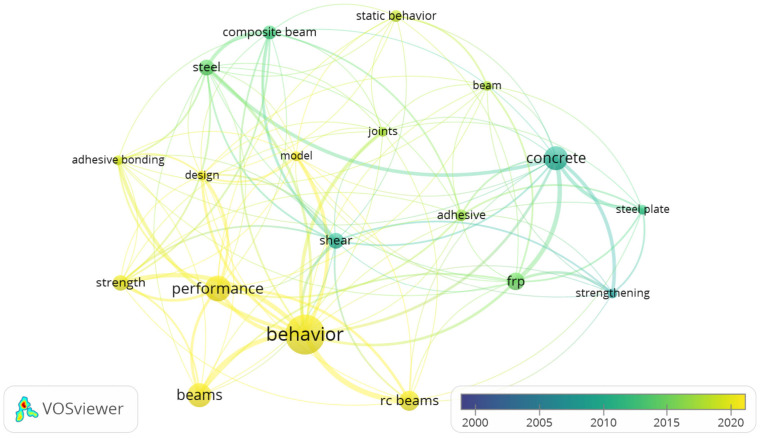
Keyword co-occurrence network and temporal evolution. Source: VOSviewer.

The keyword co-occurrence network generated with VOSviewer reveals a dense, highly interconnected research structure, indicating a thematically structured and methodologically interconnected field (Figure 5). The most prominent and central keywords, namely, behavior, performance, steel, concrete, and beams, dominate the network, demonstrating that the literature is primarily organized around structural response and mechanical assessment. The absence of isolated clusters suggests strong conceptual overlap among studies, with experimental, numerical, and analytical approaches frequently combined. Overall, the configuration reflects a thematically structured research domain centered on the evaluation of structural behavior in bonded steel–concrete systems. It should be noted, however, that keyword co-occurrence captures lexical association rather than methodological maturity, so this apparent cohesion is best interpreted as terminological convergence around structural-performance themes rather than as evidence of a fully consolidated or empirically validated field.

Three main thematic domains can be distinguished within the network. The first and most dominant concerns global structural behavior and performance, encompassing terms such as behavior, performance, beams, and strength, indicating a consistent emphasis on load-carrying capacity, stiffness, ductility, and failure mechanisms. The second domain relates to interface mechanics and adhesive bonding, linking adhesive, joints, steel and concrete, and highlighting the importance of stress transfer mechanisms and debonding phenomena in composite action. The third domain reflects the influence of fiber-reinforced polymer (FRP) strengthening research, with FRP and strengthening remaining interconnected with bond- and performance-related terms, suggesting that earlier developments in externally bonded reinforcement have significantly shaped the conceptual framework of steel–concrete bonded systems.

The temporal overlay visualization indicates a clear thematic evolution. Earlier studies, represented by cooler color tones, are predominantly associated with FRP, adhesive and concrete, reflecting an initial focus on bond mechanics and strengthening techniques. Subsequent research phases show increasing attention to composite beam modeling and parametric numerical investigations. More recent publications, represented by warmer tones, are strongly associated with behavior and performance, indicating a transition towards integrated structural response assessment at the system level. This progression reflects a shift in research focus over time, from local interface behavior towards integrated structural behavior evaluation.

Steel occupies a structurally central position within the network, acting as a conceptual bridge between adhesive interface studies and investigations of global structural behavior. Its strong interconnections with both concrete and adhesive-related terms confirm its role in integrating material-level bond mechanics with overall composite beam performance. This centrality demonstrates that bonded steel–concrete systems are framed within broader composite structural design paradigms rather than being treated as isolated material innovations.

Despite the overall density and coherence of the network, keywords related to durability, fatigue, environmental exposure, long-term degradation and lifecycle performance appear marginal or absent. This pattern suggests that the existing literature remains predominantly focused on short-term mechanical behavior and ultimate strength capacity. The limited prominence of long-term performance indicators highlights a significant research gap, particularly in relation to service-life prediction, environmental conditioning effects and probabilistic reliability assessment, which represent critical directions for future investigation in adhesively bonded steel–concrete composite systems.

#### 3.2.3. Citation Analysis of Influential Studies

A citation analysis was conducted to highlight influential contributions that have shaped the development of adhesively bonded steel–concrete composite systems. The ten most cited studies are presented in Table 5. Citation per-year counts ranged from 2.6 to 47.8 at the time of data extraction.

The research scope column distinguishes the entries concerned directly with bonded steel–concrete composite action from those belonging to the broader strengthening plate- and FRP-to-concrete bond systems. The latter, in particular the anchorage of Chen and Teng, partly accounts for the high absolute citation counts, which the per-year normalization tempers.

### 3.3. Risk of Bias in Studies

**Figure 6:**
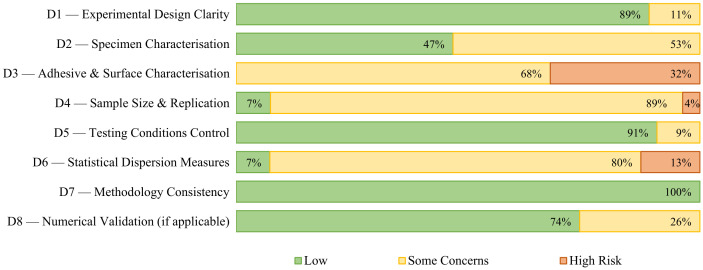
Risk of bias assessment summary result across 8 methodological domains for the 64 included studies.

The risk of bias in the included studies was assessed using the eight-domain framework described in Section 2.7, applied independently by all three authors, with disagreements resolved through discussion. The assessment produced varied findings across the defined domains, with results summarized in Figure 6. The full data for the risk of bias assessment results are available in the Supplementary Material (File S2).

In terms of experimental design clarity and internal consistency (Domains 1 and 7), most studies were classified as presenting low risk. The stated research objectives were generally consistent with the reported outcomes, and the specimen configurations were sufficiently described to allow basic replication. However, a substantial proportion of studies raised concerns about specimen characterization (Domain 2), particularly regarding the classification of steel substrate type and surface condition. In several cases, only bulk mechanical properties were reported, with no distinction between hot-rolled and cold-formed steel (CFS) substrates, no documentation of coating systems, and no characterization of surface roughness or oxide layer condition prior to bonding.

The highest risk of bias was identified in Domain 3. Transparency in reporting adhesive properties and surface preparation procedures was assessed as presenting a high risk of bias in a considerable portion of the experimental studies: adhesive mechanical properties were only partially reported in many cases, and surface preparation protocols were frequently described at a level of generality insufficient to ensure reproducibility. Reporting of statistical dispersion measures (Domain 6) was assessed as raising concerns in most of the studies and as high risk in some. Although this domain did not reach an overall high-risk judgment, the near absence of low-risk studies and the prevalence of incomplete dispersion reporting constitute a recurring limitation with direct implications for cross-study comparability.

Sample size and replication strategies (Domain 4) were a concern in most experimental investigations, with specimen counts often limited to two or three replicates per test configuration. Control of testing conditions (Domain 5), including loading protocol and environmental exposure parameters, was predominantly adequate for short-term mechanical studies but raised concerns in the subset of case study investigations, where exposure conditions were often described qualitatively rather than quantitatively. For numerical studies, validation procedures (Domain 8) were generally considered adequate when experimental data from the same study were available, but some concerns were noted when model calibration relied on previously published results without explicit uncertainty assessment.

Considering the cumulative assessment across domains, the overall risk of bias at the study level is considered moderate for the experimental corpus and low to moderate for hybrid and computational studies. No study presented systemic inconsistency between stated objectives and reported results. During the directed screening of outcome direction, 11 of the 64 included studies were identified as explicitly reporting unfavorable, neutral, or limitation-related findings alongside favorable results, including degradation-induced reductions in bond strength or residual capacity, premature debonding or brittle interfacial failure, test-method sensitivity, marginal strengthening benefits, or limited influence of specific parameters under the assessed conditions [24,28,31,32,35,37,39,43,44,54,80]. Therefore, no direct evidence of systematic suppression of unfavorable outcomes was identified within the reviewed corpus. Nevertheless, the recurring deficiencies in adhesive property documentation, surface preparation reporting, and statistical dispersion constitute identifiable sources of methodological bias that constrain the interpretability and transferability of individual findings.

To reduce bias at the review level, several measures were implemented. The eligibility criteria were defined prior to the commencement of screening, and the search strategy was applied consistently across both databases. Screening and eligibility assessment were conducted independently by the three co-authors, with disagreements resolved through discussion until consensus was reached. The findings of the risk of bias assessment were carried forward into the interpretation of results and are further discussed in relation to reporting bias patterns in Section 3.5.

### 3.4. Results of Syntheses

The thematic classification of the reviewed studies reveals a research landscape predominantly oriented toward strengthening applications and predictive modeling. Given the substantial heterogeneity across studies in experimental configurations, specimen geometries, adhesive systems, testing protocols, and reported performance metrics, a qualitative thematic synthesis was adopted rather than a statistical meta-analysis. Table 6 summarizes the distribution of the literature across the six previously defined thematic axes.

To complement the thematic classification, selected design-relevant variables were quantified from the study corpus, as summarized in Table 7. The results confirm the predominance of static monotonic testing and epoxy-based adhesive systems, while failure modes remain comparatively limited. Table 7 also reveals substantial reporting gaps in variables that directly govern interfacial behavior, particularly surface treatment and bond-line thickness. This finding is relevant because these parameters are often treated as primary research variables in adhesive bonding, yet they are not consistently documented across the reviewed corpus. Consequently, the heterogeneity identified in this review reflects not only diversity in test configurations and structural applications, but also the absence of harmonized reporting protocols. This supports the need for standardized experimental descriptions and interface-related reporting requirements before robust design-oriented comparisons can be established for adhesively bonded steel–concrete composite systems.

**Figure 7:**
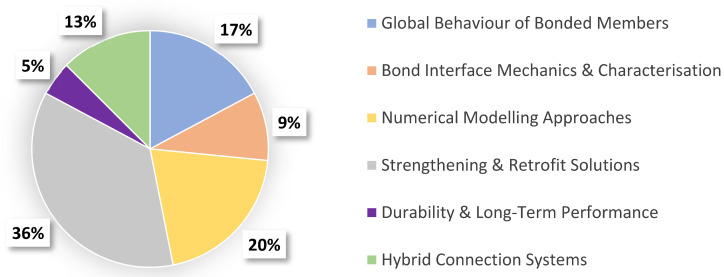
Numerical proportional distribution of studies within thematic axes.

The numerical distribution of the literature across six defined thematic axes is shown in Figure 7.

Strengthening and retrofit solutions constitute the largest thematic group, representing 36% of the reviewed studies. These investigations focus on bonded steel or cold-formed steel (CFS) elements applied to enhance or rehabilitate existing reinforced concrete members. The primary objective of this axis is to assess the efficiency of structural strengthening, the mechanisms of load transfer, and the practical applicability of externally bonded reinforcement strategies. The predominance of this theme reflects the field’s strong application-driven orientation.

Numerical modeling approaches account for 20% of the total sample. These studies are centered on FEM, parametric analysis, and predictive computational frameworks for reproducing structural or interfacial responses. Their primary objective is to develop validated numerical tools that can extend experimental findings and explore broader parametric scenarios.

Research on the global behavior of bonded members accounts for 17% of the literature. These studies evaluate shear, flexural, and overall structural behavior at the element or member scale to quantify load-bearing capacity, stiffness development, and failure mechanisms.

Bond interface mechanics and characterization comprise 9% of the reviewed works. This axis focuses specifically on interfacial shear transfer mechanisms, bond–slip relationships, failure mechanisms, and debonding prediction, aiming to characterize the constitutive laws governing interface response.

Hybrid connection systems account for 13%, with load-sharing and redundancy analyzed in systems combining adhesive bonding with discrete mechanical connectors. These studies aim to improve robustness and mitigate brittle debonding failures.

Finally, durability and long-term performance studies account for only 5% of the literature. These contributions evaluate fatigue, environmental exposure, or time-dependent degradation. The limited representation of this axis highlights a critical gap in long-term reliability assessment, particularly under service and environmental conditions.

Overall, the thematic distribution demonstrates a strong emphasis on structural strengthening and modeling, with comparatively limited attention to durability-driven performance and lifecycle considerations.

### 3.5. Reporting Biases

The qualitative assessment of reporting bias identified several recurring patterns across the included studies. Considering the predominance of an experimental methodological approach (52%) and strengthening-oriented thematics (36%), a considerable proportion of studies reported favorable structural behavior outcomes, particularly in stiffness enhancement, load-bearing capacity, and delayed crack propagation under static loading conditions. Neutral or unfavorable outcomes were comparatively underreported. Only a limited number of investigations explicitly documented premature debonding, brittle failure mechanisms, marginal strengthening benefits, or degradation-induced reductions in structural capacity.

The thematic underrepresentation of durability and long-term performance studies (5%) further indicates an imbalance in the current evidence base. While short-term strengthening efficiency is extensively documented, long-term degradation processes, fatigue behavior, and environmental aging effects remain comparatively underexplored. This asymmetry may contribute to an optimistic perception of practical performance under real service conditions.

The study-level deficiencies identified in Section 3.3, particularly the incomplete reporting of adhesive mechanical properties, surface preparation protocols, and statistical dispersion measures, are not isolated occurrences but constitute a systematic pattern across the corpus. Across the methodological categories, these omissions were most prevalent in experimental studies within the strengthening and retrofit thematic axis, where procedural details of bonding application were often treated as secondary information. This recurrent pattern limits the comparability of reported results and constrains the development of unified performance benchmarks for adhesively bonded steel–concrete composite systems.

Durability-related outcomes were frequently described qualitatively rather than supported by quantitative degradation metrics. Although environmental exposure conditions were often outlined, residual strength retention, stiffness reduction percentages, and time-dependent performance indicators were inconsistently documented. Long-term aging studies incorporating sustained environmental–mechanical coupling were scarce, and systematic monitoring over extended durations was rarely implemented.

While no direct evidence of systematic suppression of unfavorable findings was detected, the combined predominance of strengthening-focused investigations and the limited reporting of negative or degradation-driven outcomes suggests a potential publication tendency toward positive structural behavior results. This pattern was carefully considered during evidence synthesis and contributed to a more cautious interpretation of conclusions related to long-term durability and lifecycle reliability.

### 3.6. Certainty of Evidence

The certainty of evidence was assessed qualitatively for two outcome domains: short-term structural efficiency, encompassing load-bearing capacity, stiffness, and composite action under static loading; and long-term durability performance, covering environmental aging, fatigue, and time-dependent degradation. Following an approach informed by the GRADE framework [87], adapted for experimental structural engineering research, each domain was evaluated against four factors: consistency of findings across studies, methodological quality and reporting transparency, completeness of performance metrics, and homogeneity of experimental conditions. A high classification requires all four factors to be satisfactory; moderate is assigned when at least two factors are satisfactory, and none is critically deficient; low is assigned when one or more factors present critical deficiencies.

For short-term structural efficiency, certainty was classified as moderate. The consistency of findings is satisfactory, with convergent results across the majority of independent experimental studies. The remaining three factors are only partially satisfactory, reflecting incomplete reporting of adhesive properties and surface preparation (D3), systematic absence of dispersion measures (D6), and moderate heterogeneity in specimen configuration and loading protocols.

For long-term durability performance, certainty was classified as low. All four factors present critical deficiencies: the number of studies addressing extended environmental exposure is insufficient to establish convergent trends, the risk of bias in exposure characterization is high (D3, D5), performance metrics are predominantly reported qualitatively, and conditioning procedures are too heterogeneous for meaningful cross-study comparison.

## 4. Discussion

### 4.1. General Interpretation of the Results

Overall, the SLR indicates that adhesive bonding can ensure load transfer and provide composite action, not just in strengthening/retrofit systems, but also as a global connection system, especially under static loading conditions. However, the magnitude of connection performance varies considerably depending on adhesive properties, adherend characteristics, surface preparation, bond thickness, and environmental exposure conditions.

Durability-related findings reveal increasing research attention in recent years, yet long-term performance data remain critically limited. While several studies report satisfactory short-term behavior, fewer investigations systematically address aging effects, sustained loading, and aggressive environmental conditions. This indicates that conclusions regarding long-term structural reliability should be interpreted cautiously. A more detailed reading of the evidence indicates that durability is governed by several distinct degradation mechanisms that are rarely separated in the reviewed studies, namely, moisture-induced plasticization of the adhesive, steel corrosion at the interface, degradation of the concrete surface layer, thermal mismatch between adherends, viscoelastic creep of the adhesive, fatigue-driven debonding, and coupled hygrothermal and mechanical effects. Each acts through a different pathway and requires different evidence to be characterized. In practical terms, these mechanisms can be probed through hygrothermal conditioning, salt-spray testing, wet- and dry-cycle testing, sustained-load and creep testing, and cyclic fatigue protocols, and they are best captured through indicators such as residual bond strength and fracture energy, stiffness retention, interfacial slip evolution, and changes in failure mode. From a modeling perspective, representing these processes requires time-dependent, degradation-calibrated interface laws rather than the currently prevailing time-invariant formulations. Linking each mechanism to its testing protocol, measurable indicator, and modeling strategy provides a strong basis for durability-informed design.

### 4.2. Limitations of the Evidence

The body of literature analyzed presents several structural limitations that affect the field’s overall interpretability. A substantial degree of methodological heterogeneity was observed across studies, including variations in specimen configuration, adhesive systems, bond thickness, surface preparation procedures, curing conditions, and loading protocols. Such diversity limits direct comparability and constrains the identification of unified behavioral trends. In addition, reporting depth varies considerably, with inconsistencies in the documentation of adhesive mechanical properties, interfacial parameters, and environmental conditioning details. These factors indicate that, although the field demonstrates conceptual coherence, the current evidence base remains fragmented in terms of methodological standardization and comprehensive performance documentation.

The same limitations apply specifically to the numerical and analytical evidence. Because the models were developed for different specimen geometries, adhesive systems, interface laws, loading configurations, and calibration datasets, their outputs cannot be directly compared or pooled. Several models were calibrated against short-term monotonic tests, and only a small subset incorporated time-dependent, cyclic, creep, or degradation-related effects. In addition, validation depth varied across studies, with some models validated against the same-study experiments and others relying on previously published data or limited uncertainty assessment. Consequently, the numerical models are discussed as reported evidence within the reviewed studies, rather than as standardized or directly transferable predictive tools.

### 4.3. Limitations of the Review

Despite adherence to the PRISMA 2020 guidelines and the implementation of a structured and transparent review protocol, certain limitations inherent to the review process must be acknowledged.

Pre-registration practices are frequently used in clinical and biomedical systematic reviews and are not yet common in the structural engineering or construction field. According to Booth et al. [88], many platforms, including the most widely used registry (PROSPERO), explicitly restrict their scope to reviews with health-related outcomes. Among published SLRs in the health sciences that explicitly reported using PRISMA, only 38% had a registered protocol, indicating low adherence in the field for which it was specifically designed [89]. Therefore, this review was not pre-registered on a public repository before starting.

The screening and eligibility assessment were conducted independently by the authors, with disagreements resolved through discussion involving all three co-authors. However, inter-rater reliability was not formally quantified using a standardized metric. This should be considered when interpreting the thematic classification and the risk of bias assessment presented in Section 3.3.

The inclusion criteria were restricted to peer-reviewed journal articles published in English, potentially excluding relevant contributions in other languages, as well as contributions in conference proceedings or technical reports. As the review primarily focused on thematic and methodological characterization rather than quantitative meta-analysis, conclusions are based on qualitative synthesis and bibliometric patterns. Furthermore, the analysis relied on the information explicitly reported in the selected publications; therefore, any omissions or inconsistencies in the original articles may have influenced data extraction and thematic classification. These constraints were considered during interpretation and highlight the need for continued expansion and refinement of the evidence base. In particular, although a synonym-based sensitivity search was performed to test the robustness of the search strategy, a residual risk remains that studies employing uncommon terminology, or indexing their work outside the descriptors used here, may not have been fully captured. Although authorship and journal title were not used as inclusion criteria, the restriction to peer-reviewed journal articles indexed in Scopus and Web of Science may have introduced source-selection bias by excluding conference papers, theses, technical reports, and non-indexed publications.

### 4.4. Influence of Steel Substrate Characteristics on Interfacial Performance

**Figure 8:**
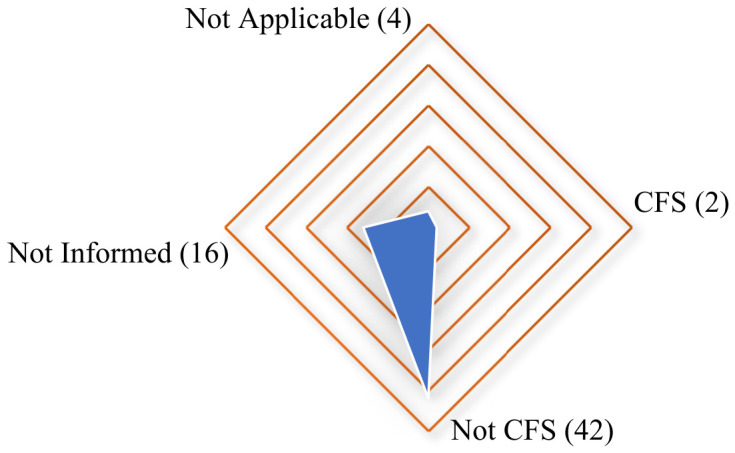
Distribution of the steel substrate types reported across the reviewed studies.

Substantial heterogeneity was identified not only in adhesive formulation and testing configuration but also in steel substrate type and surface condition. Both hot-rolled structural steel and cold-formed steel (CFS) elements are used across studies, yet their distinct surface morphologies, residual stress states, and coating systems are often not explicitly considered. To substantiate this observation, the corpus was screened for explicit reporting of steel substrate type. Of the 64 included studies, 44 studies (68.75%) explicitly reported sufficient information to classify the steel substrate, whereas 16 studies (25.0%) did not explicitly report the steel substrate type, providing only bulk mechanical properties or generic descriptions of steel elements. The remaining four studies (6.25%) were classified as not applicable for this specific substrate distinction. Among the explicitly classifiable studies, 2 (3.1% of the corpus) involved CFS substrates, while 42 (65.6%) involved non-CFS steel substrates, generally conventional steel plates or structural steel elements. These figures quantify the extent to which the steel substrate type is not explicitly reported in several cases and support the need to treat substrate type as a relevant source of variability. However, in an adhesively bonded joint, the substrate surface plays a crucial role in the joint behavior. This variability introduces a mechanistically significant source of dispersion in bond performance that is rarely treated as a primary research variable. Figure 8 quantifies the distribution of the steel substrate types reported across the evaluated studies.

Cold-formed steel, CFS, typically exhibits a smoother surface finish, a strain-hardened microstructure, and residual stresses induced during forming. In contrast, hot-rolled sections exhibit mill scale, surface roughness variability, and different oxide layer characteristics. Surface topography directly influences mechanical interlocking and effective adhesive wetting, while oxide composition affects chemical adhesion mechanisms at the steel–adhesive interface. Galvanized coatings further complicate this behavior; however, their coverage in the reviewed literature is very limited. Screening of the 64 included studies showed that only two study (3.1%) explicitly addressed galvanized or zinc-coated steel substrates, namely, the use of externally bonded galvanized steel sheets [24,77]. Zinc layers modify surface energy, corrosion resistance, and diffusion pathways, potentially altering both initial bond strength and long-term degradation kinetics. The presence of a galvanized layer may improve corrosion protection but can reduce chemical adhesion if surface preparation is inadequate, particularly under hygrothermal exposure.

From a durability perspective, substrate type influences moisture transport, corrosion initiation, and interfacial stress redistribution. In galvanized systems, differential corrosion mechanisms and interfacial debonding may be governed by zinc–adhesive interactions rather than steel–adhesive cohesion. In cold-formed thin-walled elements, reduced thickness increases sensitivity to interfacial degradation and local buckling interactions once composite action is partially lost. However, most reviewed studies treat steel as a homogeneous material, with limited differentiation among substrate categories or systematic comparisons of finishing conditions.

This lack of substrate-specific assessment constrains the transferability of experimental findings and complicates the development of durability-informed constitutive models. Interfacial parameters such as peak bond stress and fracture energy should, in principle, be defined as functions not only of adhesive properties but also of substrate roughness, coating chemistry, and surface preparation protocol. Future research should explicitly incorporate substrate surface characterization into design and degradation modeling frameworks, enabling the derivation of interface laws conditioned on steel type and surface state. Without such differentiation, predictive modeling may underestimate variability and misjudge long-term reliability.

### 4.5. Adhesive Material Properties and Their Reporting Relevance

The reviewed literature indicates that the structural response of adhesively bonded steel–concrete composite systems cannot be interpreted solely from member geometry, loading configuration, or global failure mode. The adhesive layer contributes to stress transfer at the steel–concrete interface, but its influence depends on its interaction with the substrate. Variables such as surface characteristics, bond-line thickness, curing condition, and loading regime are strictly related to adhesive bonding behavior. Therefore, adhesive properties should be treated as part of a coupled material–interface–structure system rather than as an isolated predictor of performance.

Across the reviewed corpus, epoxy-based systems are the predominant adhesive family. However, the level of information provided for the adhesive layer varies considerably. Some studies report elastic modulus, tensile or shear strength, curing conditions, and adhesive thickness, whereas others provide only the commercial product name or a generic material description. Properties such as fracture energy, glass-transition temperature, viscoelastic behavior, and moisture sensitivity are less consistently reported, despite their potential relevance for interpreting debonding, durability, and numerical model calibration.

In this context, the purpose of Table 8 is not to establish a hierarchy of adhesive properties or to imply that their effects are already quantitatively resolved for bonded steel–concrete systems. Instead, the table identifies adhesive-related descriptors that are relevant to structural interpretation and that should be reported more consistently in future studies. For example, stiffness-related properties may help interpret initial slip and connection stiffness, whereas strength-related properties are relevant to damage initiation. Fracture-related parameters are particularly important when cohesive-zone or bond–slip formulations are adopted, yet they are rarely reported in experimental studies. Similarly, thermal, moisture, and time-dependent properties may affect long-term performance, although the available evidence remains insufficient to support generalized durability factors.

Inconsistent reporting of these parameters limits the transferability of results across studies and restricts calibration of interface models. Future investigations should therefore document adhesive properties, along with a minimum of information on important properties such as substrate and surface characterization, bond-line thickness, and curing conditions. This reporting would not, by itself, eliminate the intrinsic variability of bonded steel–concrete systems, but it would provide the minimum basis required for more reliable interpretation, comparison, and modeling of structural performance.

### 4.6. Integrated Performance Framework: From Interface Mechanics to Reliability-Based Design

The synthesis of the reviewed literature shows that structural efficiency, durability, and long-term reliability in adhesively bonded steel–concrete composite systems cannot be considered separate performance domains. These aspects are inherently coupled through the behavior of the adhesive interface, which governs stress transfer and composite interaction between steel and concrete components.

Interfacial response cannot be treated as an intrinsic material constant. It depends on the material–substrate system, including steel type, coating condition, surface morphology, preparation procedures, adhesive formulation, curing regime, and bond thickness. The heterogeneity identified across the reviewed studies demonstrates that variations at this level directly affect the constitutive behavior of the interface and, consequently, the global structural response. Differences in substrate condition or adhesive properties, therefore, influence stiffness development, load redistribution, and failure mechanisms at the structural scale.

**Figure 9:**
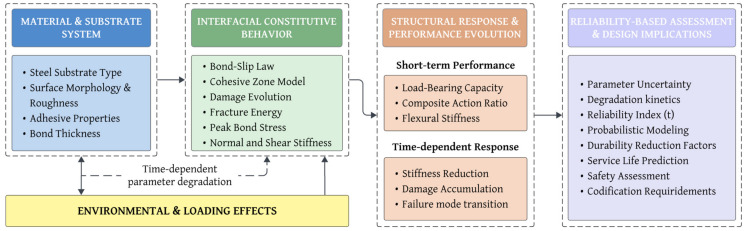
Conceptual multilevel framework linking material–substrate conditions, interfacial constitutive behavior, structural behavior evolution, and reliability-based design.

The proposed multilevel framework, illustrated in Figure 9, conceptualizes this coupling. Material and surface parameters define the constitutive behavior of the interface, including stiffness characteristics, bond strength, fracture energy, and damage evolution mechanisms. These interfacial properties control system-level indicators, including composite action development, load-bearing capacity, slip response, and failure mode transition. Structural efficiency is therefore a direct manifestation of interface mechanics.

Environmental exposure and loading regimes should be considered, as they modify this constitutive layer over time. Hygrothermal diffusion, chemical interaction, viscoelastic creep, and fatigue-induced damage progressively alter interfacial properties. The adhesive interface must therefore be understood as an evolving mechanical layer. As degradation accumulates, composite action may diminish, stiffness may reduce, and safety margins may gradually decrease under service conditions.

Despite this multilevel interaction, the modeling evidence remains dominated by time-invariant formulations. To quantify this observation, the studies containing numerical or analytical modeling were classified according to whether they used time-invariant cohesive-zone, bond-slip, finite-element, or analytical formulations calibrated mainly under short-term monotonic loading, or whether they incorporated time-dependent or degradation effects. Of the 27 modeling studies identified in the corpus, comprising 8 computational studies and 19 hybrid experimental-computational studies, 25 studies (92.6%) relied on time-invariant formulations, while only 2 studies (7.4%) incorporated time-dependent or degradation-related effects, namely, cyclic degradation/crack-propagation modeling and creep effects [63,72]. Interfacial parameters are therefore commonly treated as deterministic and constant, and degradation kinetics or variability associated with substrate condition and environmental exposure are rarely incorporated. Consequently, predictive simulations may reproduce short-term behavior with reasonable accuracy but remain limited in their ability to represent long-term behavior.

From a reliability perspective, this deterministic treatment represents a fundamental constraint. Variability in bond strength, fracture energy, and degradation rate arises from substrate heterogeneity, differences in surface preparation, adhesive dispersion, and exposure history. However, such variability is seldom characterized probabilistically. The lack of degradation-calibrated stochastic modeling and long-term experimental validation limits service-life prediction and reliability-index evaluation. This limitation has direct implications for codification. Current composite design standards, including EN 1994-1-1 [90] and AISC 360 [91] are formulated around mechanically connected systems and do not provide durability reduction factors or reliability-based criteria for adhesive-driven composite action.

The integrated framework presented in this review, therefore, identifies substrate-conditioned interfacial mechanics as the governing mechanism linking structural efficiency, durability evolution, and long-term reliability. Progress in this field requires the development of time-dependent interface laws, the statistical characterization of interfacial parameter variability, and extended validation under coupled environmental and mechanical exposure conditions. Only through this integration can adhesively bonded steel–concrete composite systems transition from an experimentally validated strengthening technique to a durability-calibrated structural design solution.

## 5. Conclusions

The PRISMA 2020-based SLR provides a structured synthesis of the scientific literature on adhesively bonded steel–concrete composite systems, highlighting their use for structural strengthening and in composite structural elements. The study integrates bibliometric analysis, thematic classification, and qualitative evidence assessment. The results demonstrate that the methodology is predominantly experimental and application-driven, with 52% of studies using experimental methods and 30% using hybrid (experimental + computational) ones.

The thematic distribution confirms that 36% of the studies were focused on strengthening and retrofit applications. Structural efficiency is primarily assessed through load-bearing capacity, stiffness enhancement, and composite action development, and short-term performance improvements are consistently reported across independent investigations. Bibliometric mapping confirms that research is conceptually centered on global structural behavior and performance, with interfacial mechanical properties playing a key role in the composite system’s response.

Despite these advances, durability and long-term performance remain the most significant unresolved challenges in the development of adhesive-bonded systems. Existing investigations represent only a limited portion of the thematic landscape and often examine degradation mechanisms at the adhesive interface. Environmental aging, fatigue behavior, sustained loading effects, and aggressive exposure conditions are rarely integrated within unified assessment frameworks, and time-dependent effects are still insufficiently incorporated into predictive modeling. As a result, although structural efficiency gains are well documented, the translation of adhesive bonding into durability-informed structural design remains incomplete. Future research must prioritize coupled environmental–mechanical aging protocols that replicate realistic service conditions, develop accelerated aging methodologies calibrated against long-term data, and advance durability-informed constitutive and probabilistic reliability models that capture material variability and degradation over time.

The analysis of the research questions further highlights critical directions for future investigation. With respect to structural efficiency assessment, there is a need to harmonize performance indicators and to develop integrated evaluation frameworks capable of linking strength, serviceability, and durability. Methodological heterogeneity across experimental studies calls for greater standardization of testing protocols and greater transparency in reporting to enhance reproducibility and comparability.

Finally, large-scale validation studies and long-term field monitoring remain scarce, limiting confidence in the actual implementation of structural measures. The establishment of durability-informed design criteria and reliability-based performance frameworks will be fundamental to support codification efforts and broader adoption in structural engineering practice. To translate these findings into actionable guidance, the following four priorities emerge.

▪ Future experimental studies should adopt a minimum reporting checklist covering adhesive mechanical properties and glass-transition temperature, bond-line thickness, surface-preparation protocol, substrate type and coating, statistical dispersion measures, and conditioning parameters, so that results become comparable across investigations.
▪ Some of the most urgent durability protocols concern coupled hygrothermal–mechanical aging, sustained loading combined with environmental exposure, and fatigue-debonding characterization, as these conditions govern realistic service behavior.
▪ The required modeling developments are time-dependent and stochastic interface laws calibrated against long-term data, replacing the prevailing time-invariant and deterministic formulations.
▪ The principal barrier to codification is the absence of durability-reduction factors and reliability-based criteria for adhesive-driven composite action in EN 1994-1-1 [90] and AISC 360 [91], which currently restrict the adoption of bonded systems as a primary structural solution.

Beyond mapping the evidence, the principal contribution of this review is a substrate-conditioned framework that links interfacial mechanics, durability evolution, and reliability-based design, together with a roadmap for the durability-calibrated criteria that current adhesively bonded steel–concrete codes do not yet provide. Realizing that transition, from an experimentally validated strengthening technique to a reliability-based structural solution, remains the central challenge for future research on adhesively bonded steel–concrete composite systems.

## Figures and Tables

**Table 1:** Selection criteria for systematic literature review (SLR).

Criteria	Inclusion	Exclusion
Publication type	Peer-reviewed journal articles (experimental, numerical/analytical, case study, or review studies).	Conference papers, theses, editorials, technical reports, working papers, and non-peer-reviewed documents.
Language	English.	Any other language.
Period	Published from 2000 to February 2026.	Publications outside the defined period.
Research scope	Studies investigating steel–concrete composite structures in which the adhesive bond is the primary or an explicitly characterized load-transfer mechanism at the steel–concrete interface.	Studies focusing exclusively on FRP–concrete systems, purely material-scale adhesive research, or non-structural adhesive applications.

**Table 2:** Operational decision rules for boundary cases during full-text screening.

Boundary Case	Decision	Governing Rule
Steel-plate strengthening of RC members	Conditional inclusion	Retained only when the adhesive bond was responsible for the shear-transfer mechanism; excluded when bonding interface was not between concrete and steel.
Hybrid FRP–steel strengthening	Conditional exclusion	Excluded unless a steel–concrete adhesive interface was explicitly characterized or validated.
Adhesive-assisted mechanical connectors	Inclusion	Retained and classified under the hybrid connection axis.
Material-scale bond tests	Conditional inclusion	Retained only when results were explicitly linked to a structural steel–concrete joint configuration.
Adhesive bonding secondary to the primary mechanism	Conditional inclusion	Applied across all categories when the bonded interface was intended to act as a load-transfer mechanism, classified under hybrid connection axis.

**Table 3:** Domain-level criteria applied in the risk-of-bias classification.

Domain	Low Risk	Some Concerns	High Risk
D1—Experimental design clarity	Design fully described, allowing independent replication.	Design described with minor omissions affecting full reproducibility.	Design incompletely described, preventing replication.
D2—Specimen characterization	Geometry and material properties fully reported.	Partial characterization, with some properties or dimensions missing.	Key properties or geometry largely absent.
D3—Adhesive and surface reporting	Adhesive mechanical properties and surface characteristics and preparation protocol fully reported.	Adhesive and surface characteristics or surface preparation information partially reported.	Adhesive properties and/or surface preparation largely unreported.
D4—Sample size and replication	Adequate replication per configuration reported (4 or more identical specimens), or strong parametric study.	Limited replication (e.g., 2–3 specimens) without justification.	Single specimen or replication not specified.
D5—Control of testing conditions	Loading and exposure conditions quantitatively defined and controlled.	Conditions described but with limited quantitative control.	Conditions described only qualitatively or inconsistently controlled.
D6—Statistical dispersion	Dispersion measures (e.g., *StD*, *CV*) reported.	Dispersion reported only partially or for selected outcomes.	No dispersion measures reported.
D7—Methodology–results consistency	Results fully consistent with the stated methodology.	Minor inconsistencies between methodology and results.	Substantial inconsistency between stated methods and reported results.
D8—Numerical validation	Models validated against independent experimental data, with uncertainty discussed.	Validation against same-study or previously published data, without uncertainty assessment.	No validation demonstrated (not applicable to purely experimental studies).

**Table 4:** Distribution of methodological axes in the reviewed literature.

Methodological Axes	Axis Description	References
Experimental	Laboratory-based investigations involving physical testing, instrumentation, and direct measurement of structural response.	[23,24,25,26,27,28,29,30,31,32,33,34,35,36,37,38,39,40,41,42,43,44,45,46,47,48,49,50,51,52,53,54,55]
Computational (numerical/analytical)	Studies based on finite element modeling (FEM), parametric simulations, or analytical formulations for predictive assessment of structural or interfacial behavior.	[56,57,58,59,60,61,62,63]
Hybrid (experimental + computational)	Investigations combining laboratory testing with numerical modeling for validation or parametric extension.	[64,65,66,67,68,69,70,71,72,73,74,75,76,77,78,79,80,81,82]
Case studies	Research involving case study, real structural applications, pilot implementations, or in-situ evaluation.	[83,84]
Reviews/conceptual analyses	Systematic reviews, state-of-the-art analyses, or conceptual frameworks synthesizing existing knowledge.	[85,86]

**Table 5:** The ten most cited studies included in the systematic review.

Authorship	Article Title	Research Scope	Times Cited (per Year)	Year
Chen, J.F.; Teng, J.G.	Anchorage strength models for FRP and steel plates bonded to concrete [60].	Strengthening and Broader adhesively bonded connection.	1194 (47.8)	2001
Adhikary, B.B.; Mutsuyoshi, H.; Sano, M.	Shear strengthening of reinforced concrete beams using steel plates bonded on beam web: experiments and analysis [34].	Strengthening and Broader adhesively bonded connection.	106 (4.1)	2000
Luo, Y.; Li, A.; Kang, Z.	Reliability-based design optimization of adhesive bonded steel–concrete composite beams with probabilistic and non-probabilistic uncertainties [58].	Steel–concrete composite.	86 (5.7)	2011
Bouazaoui, L.; Perrenot, G.; Delmas, Y.; Li, A.	Experimental study of bonded steel concrete composite structures [23].	Steel–concrete composite.	85 (4.5)	2007
Luo, Y.; Li, A.; Kang, Z.	Parametric study of bonded steel–concrete composite beams by using finite element analysis [70].	Steel–concrete composite.	80 (5.7)	2012
Kumar, P.; Patnaik, A.; Chaudhary, S.	A review on application of structural adhesives in concrete and steel–concrete composite and factors influencing the performance of composite connections [85].	Steel–concrete composite.	79 (8.8)	2017
Zhao, G.; Li, A.	Numerical study of a bonded steel and concrete composite beam [57].	Steel–concrete composite.	70 (3.9)	2008
Chen, J.F.; Yang, Z.J.; Holt, G.D.	FRP or steel plate-to-concrete bonded joints: Effect of test methods on experimental bond strength [37].	Strengthening and broader adhesively bonded connection.	65 (2.6)	2001
Dry, C.; Corsaw, M.	A comparison of bending strength between adhesive and steel reinforced concrete with steel only reinforced concrete [41].	Strengthening and broader adhesively bonded connection.	61 (2.7)	2003
Souici, A.; Berthet, J.F.; Li, A.; Rahal, N.	Behavior of both mechanically connected and bonded steel–concrete composite beams [65].	Steel–concrete composite.	48 (3.7)	2013

**Table 6:** Thematic classification of the reviewed literature.

Thematic Axis	Axis Description	Research Justification/Primary Objective	References
Global behavior of bonded members	Studies addressing flexural, shear, and overall structural behavior of bonded composite members at the element or member scale.	To quantify structural capacity, stiffness development, and failure mechanisms at the member level.	[23,36,38,41,48,50,51,53,65,76,85]
Bond interface mechanics and characterization	Research focused on interfacial shear transfer mechanisms, bond–slip relationships, and debonding behavior.	To characterize constitutive interfacial behavior governing shear transfer and debonding initiation.	[27,28,29,37,44,62]
Numerical modeling approaches	Investigations centered on finite element modeling (FEM), parametric simulations, and predictive computational frameworks.	To develop and validate computational models capable of predicting structural or interfacial response.	[56,57,58,59,61,63,66,70,72,75,78,79,81]
Strengthening and retrofit solutions	Studies examining bonded steel or cold-formed steel (CFS) elements applied to enhance or rehabilitate existing concrete members.	To assess structural enhancement efficiency and load transfer mechanisms in bonded reinforcement applications.	[25,26,30,31,32,33,34,39,40,42,45,46,47,49,52,55,73,74,77,80,83,84,86]
Durability and long-term performance	Research evaluating fatigue, environmental exposure, and time-dependent degradation of bonded systems.	To evaluate degradation mechanisms and long-term reliability under service conditions.	[24,35,43]
Hybrid connection systems	Studies analyzing systems combining adhesive bonding with discrete mechanical connectors.	To analyze load-sharing and redundancy between adhesive and mechanical connection mechanisms.	[54,60,64,67,68,69,71,82]

**Table 7:** Distribution of selected design-relevant variables extracted from the reviewed corpus.

Loading Regime		Adhesive Type		Surface Treatment		Bond-Line Thickness		Failure Mode	
Static monotonic	50	Epoxy-based.	44	Not reported.	33	Not Reported.	32	Interface debonding /Adhesive failure.	26
Cyclic /Fatigue /Dynamic	4	Not specified.	14	Mechanical abrasion /Blasting.	20	≤1.5 mm.	2	Substrate /Structural failure.	19
Sustained /Creep /Service	4	Other /Mixed families.	6	Mechanical roughening + Primer.	5	1.5–3.0 mm.	14	Mixed interface + Structural.	15
Multiple regimes	3			Degreased or Generic preparation.	4	3.0–5.0 mm.	5	Not applicable.	3
Impact	2			Not applicable.	1	>5.0 mm.	2	Other /Unclear.	1
Not applicable	1			Other /Unclear.	1	Other.	8		
						Not applicable.	1		

**Table 8:** Adhesive material properties potentially relevant to structural behavior in adhesively bonded steel–concrete composite systems.

Adhesive-Related Descriptor	Potential Relevance to Structural Response	Interpretative Limitation When Omitted
Adhesive family/chemistry	Provides the general material context of the bond layer, including expected stiffness, strength, curing behavior and environmental sensitivity.	Results cannot be clearly associated with a class of adhesive behavior, limiting comparison between studies using different commercial or generic systems.
Elastic or shear modulus	Influences initial interface stiffness, slip development, stress redistribution and stress concentration near bonded ends.	Similar bond strengths may correspond to different stiffness and slip responses, making composite-action interpretation incomplete.
Tensile/shear strength	Provides a material-level reference for damage initiation under normal or shear stress states.	Failure loads cannot be clearly related to adhesive capacity, substrate failure or interface-controlled mechanisms.
Fracture-related parameters	Relevant when debonding propagation, post-peak response or cohesive-zone/bond–slip models are considered.	Numerical models may reproduce peak load but remain weakly supported in terms of damage evolution and failure propagation.
Glass-transition temperature, *Tg*	Indicates the temperature range over which the adhesive may retain its intended mechanical behavior.	Thermal or environmental performance cannot be interpreted relative to the adhesive’s transition range.
Viscoelasticity/creep behavior	Relevant to sustained loading, long-term slip, stress relaxation and time-dependent redistribution.	Short-term stiffness or strength results may be incorrectly extrapolated to service conditions involving sustained actions.
Moisture and chemical resistance	Relevant to hygrothermal aging, aggressive exposure, residual strength and changes in failure mode.	Durability conclusions remain difficult to separate from generic aging effects, substrate degradation or interface-specific loss of adhesion.
Curing conditions	Affect the achieved adhesive properties and may contribute to variability in stiffness, strength and failure mode.	Differences between studies may reflect curing history rather than intrinsic material or interface performance.
Bond-line thickness	Influences stress distribution, deformability, defect sensitivity and the balance between cohesive and interfacial failure.	Bond performance cannot be separated from geometric effects of the adhesive layer, especially in comparisons between specimens or studies.

## Data Availability

No new data were created or analyzed in this study. Data sharing is not applicable to this article.

## Supplementary Materials

The following supporting information can be downloaded at: https://www.mdpi.com/article/10.3390/ma19132804/s1: File S1—PRISMA 2020 checklist. File S2—Full corpus risk-of-bias assessment table.
