# Supplementary material for: Adhesively Bonded Steel–Concrete Composite Structures: A Systematic Literature Review
Source: Materials (Basel). 2026 Jul 1;19(13):2804. doi: 10.3390/ma19132804 (PMC13363131; doi:10.3390/ma19132804)
Supplement: Supplementary file 1 [file materials-19-02804-s001.zip › File S2.pdf]

Risk of Bias Assessment

Each domain rated: L = Low risk | C = Some concerns | H = High risk | " " = Not applicable

Ref.	First Author & Year	Method	D1	D2	D3	D4	D5	D6	D7	D8		Overall Risk
**EXPERIMENTAL STUDIES**												
[23]	Bouazaoui et al., 2007	Experimental	L	L	C	L	L	C	L			Moderate
[24]	Rocha et al., 2026	Experimental	L	L	C	C	L	L	L			Moderate
[25]	Jabbar et al., 2016	Experimental	L	C	H	C	L	C	L			High
[26]	Barnes & Mays, 2001	Experimental	L	C	C	C	L	C	L			Moderate
[27]	Rucka, 2018	Experimental	L	C	H	C	L	C	L			High
[28]	Zhao et al., 2019	Experimental	L	L	C	C	L	C	L			Moderate
[29]	Chataigner et al., 2011	Experimental	L	L	C	L	L	L	L			Low
[30]	Rakib & Morshed, 2023	Experimental	L	C	H	C	L	C	L			High
[31]	Li et al., 2000	Experimental	C	C	C	C	L	H	L			High
[32]	Ngidi & Dundu, 2018	Experimental	L	C	H	C	L	C	L			High
[33]	Zhang et al., 2024	Experimental	L	C	C	C	L	C	L			Moderate
[34]	Adhikary et al., 2000	Experimental	C	C	H	C	L	H	L			High
[35]	Tadeu & Branco, 2000	Experimental	C	C	C	C	C	H	L			High
[36]	Bruno et al., 2021	Experimental	L	L	C	C	L	C	L			Moderate
[37]	Chen et al., 2001	Experimental	L	C	C	C	L	C	L			Moderate
[38]	Yang et al., 2024	Experimental	L	L	C	C	L	C	L			Moderate
[39]	Ciampa et al., 2023	Experimental	L	L	C	L	L	L	L			Low
[40]	Aykac et al., 2012	Experimental	L	C	H	C	L	C	L			High
[41]	Dry & Corsaw, 2003	Experimental	C	C	H	C	C	H	L			High
[42]	Uy, 2002	Experimental	L	C	H	C	L	C	L			High
[43]	Yoshitake et al., 2016	Experimental	L	C	C	C	L	C	L			Moderate
[44]	Zima & Kedra, 2020	Experimental	L	C	H	C	L	C	L			High
[45]	Yan et al., 2023	Experimental	L	L	H	C	L	C	L			High
[46]	Atmajayanti et al., 2025	Experimental	L	C	H	C	L	C	L			High
[47]	Barnes & Mays, 2006a	Experimental	L	C	C	C	L	C	L			Moderate
[48]	Seleem et al., 2025	Experimental	L	L	C	C	L	C	L			Moderate
[49]	Minnaugh & Harries, 2009	Experimental	L	C	C	C	L	C	L			Moderate
[50]	Biscaia et al., 2018	Experimental	L	L	C	C	L	C	L			Moderate
[51]	Li et al., 2025	Experimental	L	L	C	C	L	C	L			Moderate
[52]	Zhou et al., 2024	Experimental	L	C	C	C	L	C	L			Moderate
[53]	Shao et al., 2025	Experimental	L	L	C	C	L	C	L			Moderate
[54]	Alam et al., 2016	Experimental	L	C	H	C	L	C	L			High
[55]	Thamrin et al., 2023	Experimental	L	C	H	C	L	C	L			High
**NUMERICAL / ANALYTICAL STUDIES**												
[56]	Kumar et al., 2023	Numerical	L	L	C				L	L		Low
[57]	Zhao & Li, 2008	Numerical	L	C	C				L	C		Moderate
[58]	Luo et al., 2011	Numerical	L	L	C			L	L	L		Low
[59]	Daouadji et al., 2025	Analytical	L	C	C				L	C		Moderate
[60]	Chen & Teng, 2001	Analytical	L	C	C				L	C		Moderate
[61]	Jurkiewiez et al., 2014	Numerical	L	L	C				L	L		Low
[62]	Bhardwaj et al., 2021	Numerical	L	C	C				L	L		Moderate
[63]	Bocciarelli, 2021	Numerical	L	C	C				L	L		Moderate
**HYBRID STUDIES (Experimental + Numerical)**												
[64]	Luo & Wu, 2022	Hybrid	L	L	C	C	L	C	L	L		Moderate
[65]	Souici et al., 2013	Hybrid	L	L	C	L	L	C	L	L		Moderate
[66]	Li et al., 2023	Hybrid	L	L	C	C	L	C	L	L		Moderate
[67]	Zhou et al., 2025	Hybrid	L	L	C	C	L	C	L	L		Moderate
[68]	Branco et al., 2003	Hybrid	C	C	C	C	C	H	L	C		High
[69]	Ogura et al., 2018	Hybrid	L	C	H	C	L	C	L	C		High
[70]	Luo et al., 2012	Hybrid	L	L	C	C	L	C	L	L		Moderate
[71]	Liu et al., 2025	Hybrid	L	L	C	C	L	C	L	L		Moderate
[72]	Li et al., 2025	Hybrid	L	L	C	C	L	C	L	L		Moderate
[73]	Wang et al., 2024	Hybrid	L	C	H	C	L	C	L	C		High
[74]	Wojtczak et al., 2020	Hybrid	L	C	H	C	L	C	L	C		High
[75]	Xiong et al., 2018	Hybrid	L	L	C	C	L	C	L	L		Moderate
[76]	Jing et al., 2024	Hybrid	L	L	C	C	L	C	L	L		Moderate
[77]	Hamoda et al., 2025	Hybrid	L	L	H	C	L	C	L	L		High
[78]	Yan et al., 2023	Hybrid	L	L	H	C	L	C	L	L		High
[79]	Jurkiewiez et al., 2011	Hybrid	L	L	C	C	L	C	L	L		Moderate
[80]	Xu et al., 2024	Hybrid	L	L	C	C	L	C	L	L		Moderate
[81]	Salah et al., 2024	Hybrid	L	C	C	C	L	C	L	L		Moderate
[82]	Xu et al., 2024	Hybrid	L	L	C	C	L	C	L	L		Moderate
**CASE STUDIES**												
[83]	Lopez et al., 2007	Case Study	C	C	H	H	C	H	L			High
[84]	Atea, 2019	Case Study	C	C	H	H	C	H	L			High
**REVIEW / CONCEPTUAL STUDIES**												
[85]	Kumar et al., 2017	Review	L						L			Low
[86]	Barnes & Mays, 2006	Review	L						L			Low
